# Mechanobiology of the Human Intervertebral Disc: Systematic Review of the Literature and Future Perspectives

**DOI:** 10.3390/ijms24032728

**Published:** 2023-02-01

**Authors:** Alberto Ruffilli, Giovanni Viroli, Simona Neri, Matteo Traversari, Francesca Barile, Marco Manzetti, Elisa Assirelli, Marco Ialuna, Fabio Vita, Cesare Faldini

**Affiliations:** 1 Department of Biomedical and Neuromotor Science-DIBINEM, 1st Orthopaedic and Traumatologic Clinic, IRCCS Istituto Ortopedico Rizzoli, University of Bologna, Via Giulio Cesare Pupilli 1, 40136 Bologna, Italy; 2 Medicine and Rheumatology Unit, IRCCS Istituto Ortopedico Rizzoli, Via Giulio Cesare Pupilli 1, 40136 Bologna, Italy; 3 Department of Biomedical and Neuromotor Science-DIBINEM, University of Bologna, Via Giulio Cesare Pupilli 1, 40136 Bologna, Italy

**Keywords:** mechanobiology, mechanotransduction, degenerative disc disease, intervertebral disc

## Abstract

Low back pain is an extremely common condition with severe consequences. Among its potential specific causes, degenerative disc disease (DDD) is one of the most frequently observed. Mechanobiology is an emerging science studying the interplay between mechanical stimuli and the biological behavior of cells and tissues. The aim of the presented study is to review, with a systematic approach, the existing literature regarding the mechanobiology of the human intervertebral disc (IVD), define the main pathways involved in DDD and identify novel potential therapeutic targets. The review was carried out in accordance with the Preferential Reporting Items for Systematic Reviews and Meta-analyses (PRISMA) guidelines. Studies were included if they described biological responses of human IVD cells under mechanical stimulation or alterations of mechanical properties of the IVD determined by different gene expression. Fifteen studies were included and showed promising results confirming the mechanobiology of the human IVD as a key element in DDD. The technical advances of the last decade have allowed us to increase our understanding of this topic, enabling us to identify possible therapeutic targets to treat and to prevent DDD. Further research and technological innovations will shed light on the interactions between the mechanics and biology of the human IVD.

## 1. Introduction

Low back pain (LBP) is an extremely common condition worldwide, with an expected lifetime-prevalence rate of 80% [1] and severe socioeconomic and health care consequences [2]. Among its potential specific causes, degenerative disc disease (DDD) is one of the most frequently observed [3]. On a morphological level, DDD results in dehydration, fissuring and tearing of the nucleus pulposus (NP), annulus fibrosus (AF) and endplate [4]. 

However, as opposed to being a passive process of wear and tear, disc degeneration is an abnormal, cell-mediated response to structural failure brought on by aging and other environmental variables including abnormal mechanical stress. In fact, substantial degenerative changes also happen at a biological level. A decrease in the diffusion of oxygen, nutrients and waste products through the disc space is seen [5], with a consequent shift towards an anaerobic metabolism, with increased lactate production and a decrease in pH level. The intervertebral disc (IVD) pH ranges from 7.1 in healthy tissue to 6.8 in degenerated tissue, with values reaching pH 5.5 in severely degenerated discs [6,7]. Concerning physiological loading forces acting within human lumbar IVDs, the values range.

Between 0.5 and 1.7 MPa during relaxed standing and lifting a 20 Kg weight with flexed knees, respectively. Concerning the strain stress of human annulus fibrosus in vivo, the values range between 4% and 6% when discs are in compression or torsion and flexion or extension, respectively [8,9,10].

Alongside this, while the normal IVD is avascular and aneural, micronervous [11] and microvascular [12,13] ingrowth is seen in degenerated discs. Moreover, increased cellular apoptosis [14], cellular senescence [15], cell density [16] and the substitution of notochordal cells of the NP with chondrocyte-like cells [17] occur.

Furthermore, substantial changes are seen in the extracellular matrix (ECM). A quantitative decline in the proteoglycan (PG) content occurs, as well as a deterioration of qualitative ECM composition, with decreased aggrecan content and a change in the relative proportions of the glycosaminoglycans in favor of keratan sulfate over chondroitin sulfate [18]. As a result, less water molecules can be bonded by the matrix. Collagen production patterns and compositions within the degenerated disc change: an increase in collagen I appears in the NP and in the inner AF (in which collagen II is normally predominant), while an increase in collagen II appears in the outer AF (in which collagen I is normally predominant).

Finally, degenerated IVDs show a pro-inflammatory microenvironment, as the expression of IL-1β, TNF-α [19,20], Nitric Oxide (NO), prostaglandin E_2_, IL-6 and matrix metalloproteinases (MMPs) [21,22] are significantly increased compared to controls. Recent studies further demonstrated that axial loading and mechanical stress correlated with CGRP and mPGES-1 [23]. On the first instance, this pro-inflammatory microenvironment plays a crucial role as a “discogenic pain” generator [24]. Secondly, inflammation contributes to the increased degradation of the ECM and to PG loss from the NP, with a consequent decrease in weight-bearing capacity and, eventually, leading to the aforementioned structural degenerative changes, thus establishing a vicious circle.

Mechanobiology is an emerging science studying the interplay between mechanical stimuli and the biological behavior of cells and tissues. The human spine is a complex biomechanical model constantly subjected to mechanical loading, and the IVD is the keystone of spine physiology. Given the important effect that mechanical loadings have on the IVD, it is crucial to understand the molecular pathways that, from loading, lead to DDD, in order to prevent and treat disc degeneration. There is a growing body of literature focusing on IVD mechanobiology; however, most of the studies just focus on one single molecular pathway or on a single form of mechanical stress. Some literature reviews [25,26,27,28,29] were conducted to harmonize the results achieved by the most important mechanobiological studies on IV, but none are systematic, with a consequent increased risk of selection bias. Moreover, many of the existing reviews included both human and animal models, providing a possible additional source of bias, since there are substantial differences between the physiology and the forces acting on animal and human IVDs, given their erect posture. Hence, the aim of the presented study is to review, with a systematic approach, the existing literature regarding the mechanobiology of the human IVD, defining the main pathways involved in DDD to identify novel potential therapeutic targets.

## 2. Material and Methods

### 2.1. Review Design

A systematic review of the literature regarding the mechanobiology of the human intervertebral disc was carried out following the Preferential Reporting Items for Systematic Reviews and Meta-Analyses (PRISMA) guidelines [30].

Only peer-reviewed publications were considered. Studies were included if they described biological responses of human IVD cells under mechanical stimulation or alterations of mechanical properties of the intervertebral disc determined by different gene expressions. Only articles in English were included. Articles that analyzed samples retrieved from animals or both animals and humans were excluded.

The included articles met the PICO criteria for systematic reviews (Population, Intervention, Comparison, and Outcomes).

### 2.2. Search Strategy

Eligible studies were searched in Pubmed-MEDLINE, Embase Biomedical Database, Google Scholar, and The Cochrane Central Registry of Controlled Trials between 1990 and 2022. Three reviewers (G.V., M.T. and M.M.) performed the literature search in October 2022.

The search string used included the following items: mechanobiology OR mechanotransduction OR (mechanics OR overload OR load) AND (genes OR gene OR genome OR epigenetic OR genetic OR pathway) AND (“intervertebral disc” OR “nucleus pulpous” OR “annulus fibrosus” OR “intervertebral discs” OR “intervertebral disc degeneration” OR “degenerative disc disease” OR “IVD” OR “DDD”).

### 2.3. Study Selection

**Figure 1:**
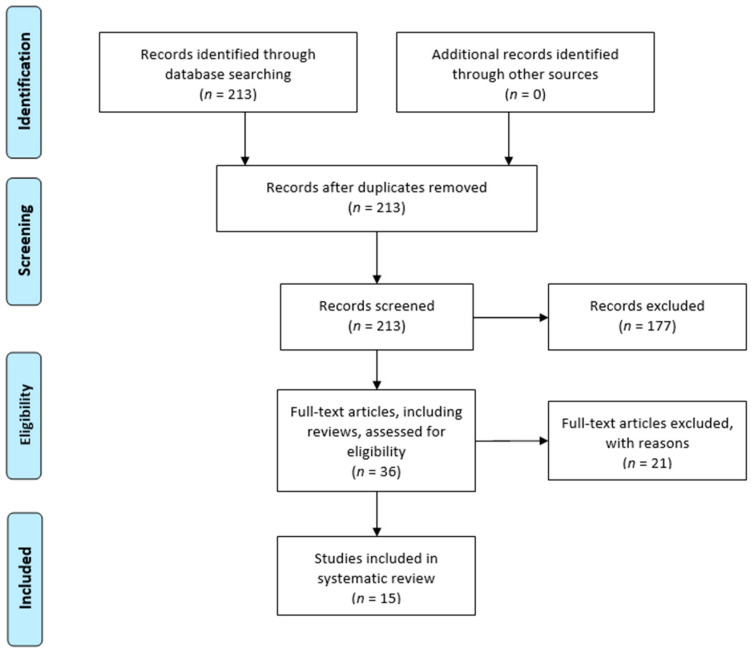
Prisma 2009 flow diagram of the included studies.

Titles and abstracts were screened, and then, relevant articles obtained via an electronic search were retrieved in full-text, followed by a hand check of their bibliography in order to recover additional related articles. This search was extended to reviews and meta-analyses for the identification of potentially missed eligible papers. Duplicates were subsequently removed. In Figure 1, the selection process carried out in accordance with the PRISMA flowchart is represented. This systematic review was registered in the PROSPERO database for systematic reviews [31] (ID: CRD42022371280).

### 2.4. Data Extraction

Two reviewers (G.V. and M.T.) extracted the data through a standardized data collection form. Three reviewers (G.V., M.T. and S.N.) checked the data for accuracy, and inconsistent results were analyzed for discussion. The extracted data concerning the study design, number of samples, demographics of patients, type of IVD cells considered for analysis, model of mechanical stimulation, cellular response to mechanical stimulation and results are summarized in Table 1.

## 3. Results

### 3.1. Included Studies

A total of 213 studies were found through the electronic search, according to the research performed. A total of 15 papers [32,33,34,35,37,38,39,40,41,42,43,44,45,46] met the inclusion criteria and were considered for review. All of the included studies were in vitro or ex vivo studies.

The following studies were not included in the final analysis: research using animal models, finite element analyses, studies that did not evaluate mechanical stimuli on samples and review articles.

The studies analyzed mainly small-sized groups of samples (n = 3 to 30), describing the relationship between mechanical stimuli and the biology of human IVD cells and the mechanical properties of human IVD cells under different biological conditions.The included studies were largely heterogeneous in terms of patients’ characteristics, the degeneration degree of different samples, gender, the type of applied mechanical stimuli, the analysis technique and the analyzed molecular pathway (Table 1).

### 3.2. Cohort Characteristics

The included studies reported data on a total of 212 IVD samples derived from 126 patients who underwent spinal surgery for DDD, 38 patients who underwent spinal surgery for idiopathic scoliosis, 14 patients who underwent spinal surgery for spinal cord injury and 30 cadavers. One study did not report accurate information about the sample origin [37].

The studies included 62 males (58%) and 45 females (42%). Seven studies did not report the patients’ sex [33,37,40,41,42,43,44]. The mean age of the patients ranged from 56.2 to 15.2 years. No study reported accurate information about patients’ ethnicity.

### 3.3. Evaluated Cell Types and Molecular Pathways

Six studies evaluated the biological response of NP cells isolated from IVD components and cultured before mechanical stimulation [32,35,36,37,44,46]. Five studies evaluated the biological response of AF cells isolated from IVD components and cultured before mechanical stimulation [34,37,38,39,41]. Three studies evaluated the biological response of both isolated NP and AF cells to mechanical stimulation [34,38,45]. Sun et al. [33] evaluated the anti-angiogenic role of exosomes obtained via the in vitro mechanical stimulation of isolated and cultured notochordal cells. Aladin et al. [45] analyzed the relationship between the mechanical properties of frozen NP tissue explants and type IX collagen variant typing.

**Figure 2:**
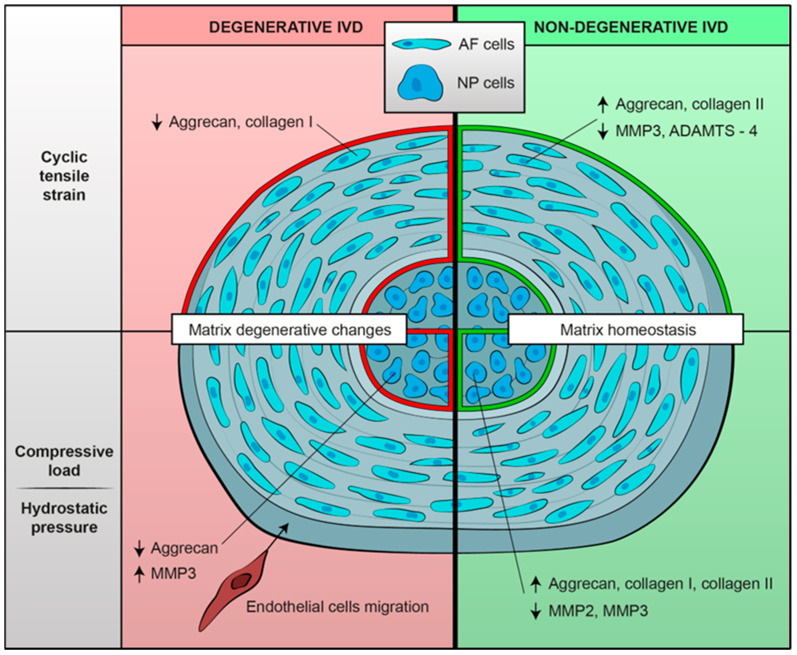
Summary of biological mechanoresponses of human intervertebral cells, either degenerative or non-degenerative, to different mechanical stimuli in terms of extracellular matrix changes. Mechanical load seems to determine a shift towards an anabolic phenotype in non-degenerated IVD cells; on the contrary, degenerated IVD cells exposed to mechanical stimulation show matrix catabolic changes.

As for molecular pathways, most of the studies analyzed the expression of catabolic and anabolic genes in IVD cells under different types and conditions of mechanical stimulation [34,35,36,41,42,43,44,45,46]. Two studies focused on the role of ion channels in mechanotransduction [38,39]. Song et al. [32] evaluated the role of the RhoA/ROCK2–MRTF-A signaling pathway in the development of the IDD. Sun et al. [33] evaluated the role of the Wnt/b-catenin axis in the inhibition of miR-140-5p. Likhitpanichktul et al. [37] investigated the interaction between TNF-α and tensile strain in IVD degeneration. Some of the key findings are summarized in Figure 2. 

### 3.4. Applied Mechanical Stimuli and Cell Environmental Conditions

The included studies used different types of mechanical stimulation in order to produce a biological response in different types of cells. It is noteworthy that in a physiological situation (in vivo), IVD cells were subjected to different loads depending on their anatomical position, with NP and inner AF cells being mainly subjected to compressive loading, while outer AF cells were subjected to strain forces [47]. Most of the studies applied cyclic tensile strain [32,34,39,40,42,43,45]. Studies focusing on NP cells mainly applied compressive load [33,36,37,44,46]. Few studies exposed cells to hydrostatic pressure [34,35,45]. Franco-Obregon et al. simulated microgravity using a random positioning machine and analyzed isolated and cultured IVD cells’ biological responses [35].

#### 3.4.1. Cyclic Tensile Strain (CTS)

Authors applied different strain protocols to study cellular responses (Table 2). Most of the experiments used a strain frequency of 1 Hz, which is similar to physiological human motion [34,39,41,42,43,45,47]. The strain magnitude varied, ranging from 1% [46] to 20% [39,43].

Neidlinger-Wilke at al. [46] found that CTS has an anabolic effect on AF cells, with the increased gene expression of aggrecan and collagen II and decreased MMP-3 gene expression. No difference was found by applying different strain magnitudes (1, 2, 4 and 8% strain). Mechanically stimulated cells tended to decrease the mRNA expression of MMP-2 and MMP-3. It is important to notice that cells obtained by different donors showed high interindividual variations in cellular responses to mechanical stimuli, suggesting that different degrees of disc degeneration could affect mechanotransduction pathways in a similar manner.

Gilbert et al. [41] investigated the effect of CTS on AF cells derived either from degenerated or healthy IVDs. Interestingly, the response appeared to be frequency-dependent and strongly dependent on the degree of disc degeneration. At 1 Hz CTS, AF cells derived from non-degenerative IVDs showed a shift to a less catabolic phenotype, decreasing ADAMTS-4 and MMP-3 expression. Conversely, AF cells exposed to 0.33 Hz CTS showed a catabolic response. AF cells derived from degenerative tissues appeared to have lost their ability to reduce metalloproteinase gene expression and also decreased matrix protein gene expression (aggrecan and collagen I). Subsequently, Gilbert et al. [39] observed that the reduction in the catabolic response in non-degenerative AF cells exposed to 1 Hz CTS may be IL-1- and IL-4-dependent. In AF cells derived from degenerative IVDs, neither IL-1 nor IL-4 showed a significant role in the matrix remodeling mechanoresponse, confirming their loss of response to stimuli.

AF cells derived from degenerative and non-degenerative tissue display strong differences in the regulation of matrix protein and matrix-degrading enzyme gene expression. Those differences could be attributed to mechanotransduction pathways altered in degenerated tissues [38,39,41]. Integrins have been shown to play a pivotal role in the mechanoresponse of AF cells to CTS [38]. In non-degenerative AF cells exposed to 1 Hz 10% CTS, the addition of an integrin-recognizing RAD peptide determined the upregulation of ADAMTS-4 gene expression and increased FAK phosphorylation. The addition of integrin-function-blocking peptide to non-degenerative AF cells exposed to 1 Hz 10% CTS was able to inhibit CTS-induced FAK phosphorylation and to upregulate ADAMTS-4 gene expression. AF cells derived from degenerative IVDs did not show integrin-dependent differences in mechanoresponses. These findings suggest that the involvement of RGD integrins in mechanoresponse is lost with degeneration; thus, integrins may not be involved in degenerative AF cells’ altered mechanoresponse, which seems to happen via an alternative mechanotransduction pathway.

Likhitpanichkul et al. [37] further evaluated the role of inflammation in matrix homeostasis and the mechanoresponse of AF cells subjected to CTS, showing that the concomitant exposure of AF cells to TNF-α and pathological strains determined increased IL-1β and IL-8 production and a modified AF cytoskeleton. These results suggested that TNF-α induced cytoskeletal changes in AF cells, suggestive of enhanced mechanosensitivity, having a detrimental effect on IVD matrix homeostasis.

Cambria et al. [34] investigated TRPV4 ion channel signaling in stretch-induced AF cells’ inflammation. In addition, they established a novel model of acute pro-inflammatory response to hyperphysiological stretching typical for early-stage AF injury (increased gene expression of IL-6, IL-8, COX2 and MMP-1 and reduced expression of collagen II and aggrecan). These findings showed that TRPV4 pharmacological inhibition and TRPV4 gene knock-out via CRISPR-Cas9 gene editing effectively mitigated the inflammation caused by hyperphysiological cyclic stretching, thus demonstrating that TRPV4 may play a role in stretch-induced inflammation, possibly via the activation of the p38 MAPK pathway.

Unlike other studies, Song et al. [32] evaluated NP cells exposed to cyclic strain, focusing specifically on the RhoA/MRTF-A signaling pathway and its role in DDD development. They applied CTS with or without an MRTF-A inhibitor and observed that the RhoA/MRTF-A signaling pathway is of key importance in NP cells’ strain-induced matrix degeneration. MRTF-A inhibition may alleviate the fibrosis caused by mechanical overload, with a potential therapeutic effect.

#### 3.4.2. Compressive Load

The differences in mechanoresponses between degenerative and non-degenerative IVD cells have been further investigated by Le Maitre et al. [43]. The compressive load of degenerative and non-degenerative NP cells seeded in alginate constructs appeared to have subtle differences in terms of mechanoresponse, determining decreased aggrecan expression. When exposed to an RGD integrin ligand site inhibitor, degenerative NP cells continued to show decreased aggrecan expression, while non-degenerative NP cells under the same loading conditions did not show load-induced aggrecan downregulation, revealing an integrin-mediated anti-catabolic effect. Thus, the differences in cellular response to mechanical load between degenerative and non-degenerative cells may be due to the altered mechanotransduction pathway. In non-degenerative NP cells, mechanotransduction seems to occur via integrin receptors, while degenerative NP cells might use different receptors.

Huang et al. [40] showed that a dynamic compressive load applied to NP tissue determined matrix enzymes’ (metalloproteinases and ADAMTSs) upregulation, leading to a decrease in aggrecan content in the NP cell matrix, therefore being an etiological factor in the development of DDD. Interestingly, ADAMTS-1, 4, 9, 15, and TIMP-3 are also physiologically expressed in healthy disc matrixes, suggesting that these proteins may play a role in regular IVD matrix turnover [40]. TIMP-3 demonstrated the potential capability to prevent matrix catabolism and to determine an increase in aggrecan expression [40].

In order to better understand the importance of the cell microenvironment, Hodson et al. [36] tested NP cells embedded in agarose constructs by applying cycling compressive loads under different pH conditions, representative of degenerative (acidic pH) and non-degenerative (neutral pH) IVD microenvironments. The compression response of NP cells at a pH representative of degenerate tissue (pH 6.5) was represented by a metabolism shift to matrix catabolism. The progression of IVD degeneration may therefore recognize acidity-induced aberrant mechanotransduction as a potential pathologic mechanism. In addition, Hodson et al. investigated whether mechanotransduction might be integrin-dependent under various pH settings, finding that aggrecan expression was modulated by integrins at a pH representative of non-degenerative IVD, while the mechanoresponsiveness of aggrecan was aborted at an acidic pH [36].

The potential role of notochordal cells’ products obtained by notochordal cells’ compression in preventing IVD angiogenesis was evaluated by Sun et al. [33]. Exosomes derived from compressed notochordal cells showed a strong inhibitive angiogenesis effect, demonstrating that exosomes are effective carriers in transferring miRNA from cell to cell. The anti-angiogenetic effect seems to occur through the inhibition of the Wnt/b-catenin pathway.

#### 3.4.3. Hydrostatic Pressure

Consistent with aforementioned results, Neidlinger-Wilke et al. [46] also showed that hydrostatic pressure, if applied in protocols similar to physiological loads, determines a shift to a less catabolic metabolism of both NP and AF cells embedded in collagen gel.

These findings might suggest that moderate mechanical loading can prevent disc matrix degradation by inhibiting MMPs’ expression.

Most importantly, for the first time, it has been assumed that the strong interindividual variations in gene expression among donors could be due to the degree of disc degeneration.

Subsequent studies [44] showed that NP cells derived from healthy IVD tissue exposed to dynamic hydrostatic pressure responded by upregulating the gene expression of anabolic genes, while NP cells derived from degenerative IVDs exposed to the same stress did not show any effect on gene expression.

#### 3.4.4. Microgravity

To elucidate the effect of reduced load on IVD cells, Franco-Obregon et al. [35] simulated microgravity using a random positioning machine and tested the mechanoresponse of both AF and NP cells in the presence or absence of TRPC6 channel pharmacological inhibition. Surprisingly, cells subjected to simulated microgravity or to TRPC channel inhibition showed reduced proliferation and increased senescence.

## 4. Discussion

In the present work, we systematically reviewed the available literature from 1990 to the present date regarding the mechanobiology of the human IVD. Fifteen papers met the inclusion criteria, exploring multiple aspects of the mechanobiology of the human IVD and showing promising results in terms of the definition of molecular pathways potentially involved in the development of DDD. Many of the included studies evaluated the mechanical response of IVD cells to different mechanical stimuli in terms of matrix homeostasis. In detail, Neidlinger-Wilke [46] demonstrated that moderate mechanical stimulation, both by HP and CTS, determines an anabolic shift of the matrix homeostasis, with some differences between the NP cells (more responsive to HP) and the AF cells (more responsive to CTS). As both hyperphysiological stresses and mechanical unloading are reported to determine a catabolic shift in the IVD [40,43,48], moderate mechanical loading may be important in preventing disc matrix degradation The clinical reflection of this is that moderate exercise may be have a protective role against disc degeneration [49]. However, this does not seem to be true for both degenerated and non-degenerated IVD. In fact, physiological HP only seems to increase the expression of collagen II and aggrecan in healthy NP cells and not in degenerated ones [44]; on the other hand, the application of 20% CTS at 1 Hz on degenerated AF cells not only fails to downregulate matrix proteases’ gene expression, but it also results in reduced aggrecan and collagen I gene expression [41]. In this light, in a clinical view, moderate physical exercise on extracellular matrix metabolism in a degenerated disc may have detrimental consequences, although this requires further research.

The reasons for these different biological responses to mechanical stimulation between degenerated and healthy discs have long been researched. Some of the included studies [36,41,46] have demonstrated that RGD-recognizing integrins lose their role in the mechanoresponse of human NP and AF cells derived from degenerated IVD compared to non-degenerated IVD cells. Since both degenerated and non-degenerated IVD cells express RGD-binding integrins, these data suggest that, with DDD, either the RGD integrin transduction is altered or that mechanosensing could occur through a non-RGD integrin. Local inflammation has also been demonstrated to be crucial in disc mechanobiology. In fact, while non-degenerated AF cells subjected to CTS showed a reduced catabolic response which appeared to be IL-1- and IL-4-dependent, in degenerated AF cells, neither IL-1 nor IL-4 appeared to be necessary for the matrix remodeling mechanoresponse [39]. Moreover, an interesting finding is that local inflammation mediated by TNF-α induces deep cytoskeletal changes, enhancing the mechanosensitivity of cells and thus creating a vicious mechanoinflammatory system at the intracellular level [37]. This inflammatory microenvironment is critical not only in the vicious loop that leads to DDD, but also in the genesis of LBP. In fact, LBP is an extremely heterogeneous symptom, and it is unclear why some degenerative discs are responsible for LBP while others are not. However, nerve in-growth in the NP induced by inflammatory cytokines [50,51] undoubtedly plays a role in the development of discogenic pain.

The relationship between mechanical stimuli, inflammation and the progression of degenerative disc disease is still under investigation. Unfortunately, many studies on this topic involved animal models and were therefore excluded. We hope that further studies will be conducted in the future.

Cambria et al. [34] demonstrated that part of this mechanoinflammation in AF cells is signaled through TPRV4 ion channels, making them a possible future pharmacological or gene-editing therapeutic target. Furthermore, other potential therapeutic targets have been identified by some of the included studies. Song et al. [32] demonstrated that the RhoA/MRTF-A inhibitor CCG-1423 can play a potential role as a therapeutic agent, since it is able to stop a signaling pathway leading to ECM degradation in response to NP cells’ mechanical overload. Sun et al. [33] demonstrated that miR-140-5p, contained in NC-exos induced by compressive load, had a significant inhibitive effect on angiogenesis in endothelial cells. This may have a therapeutic role, since mechanical load seems to have a proangiogenetic effect in the IVD [42]. Finally, Huang et al. [40] suggested that TIMP-3, an ADAMT inhibitor, may have a potential role in the prevention of aggrecan loss during DDD.

It is important to note a significant trend towards increasing complexity in study designs and techniques employed in more recent studies compared to older studies. For example, Neidlinger-Wilke [46] and Le Maitre [44] mainly conducted gene expression studies based on classical semi-quantitative RT-PCR, while Song [32] and Sun [33] reached interesting results thanks to the adoption of modern biotechnologies such as CRISPR-Cas9 genome editing and exosome characterization, respectively.

To the best of our knowledge, this is the first systematic scoping review only focusing on the mechanobiology of human IVD. This is crucial, since the results of animal mechanobiological models of IVD may not be transposed to human IVD cells [52]. In fact, the vast majority of animal models involved quadrupeds, whose spine develops in an extremely different biomechanical environment compared to the human spine, which is almost constantly exposed to the action of gravity after the assumption of an erect posture during childhood. The presented study does not come without limitations. Firstly, high heterogeneity among the included studies was registered, especially in terms of the type of cells (AF and/or NP), type of mechanical stimulation (cyclic tensile strain, hydrostatic pressure, compressive load and simulated microgravity), mechanical stimulation protocol (intensity, frequency and duration of the stimuli that are known as crucial factors in determining biochemical cell response) and type of culture environment (2 or 3D). Secondly, many studies adopted disc samples from AIS patients [33,37,44,45] or cadavers [42,46] as controls, while both the categories may show unrecognized subtle alterations in the IVD (the first, due to the underlying condition; the second, due to advanced age). In addition, only one [32] of the included studies reported data regarding systemic diseases (cardiovascular, pulmonary or neoplastic). Finally, all the included studies were based upon in vitro (almost all) or ex vivo models, which are useful in order to explore cellular mechanisms, but their findings need to be validated at the IVD organ scale and in vivo, since cellular phenotype significantly changes during in vitro culture compared to the in vivo one.

Further in vitro studies, with a more standardized stimulation model, as well as organ-scale and in vivo studies, are needed in the forthcoming years to confirm the role of the promising pathways that have been identified to have a potential role in the mechanobiology of DDD. Alongside that, future preclinical and clinical studies investigating the effects of the targeted pharmacological inhibition of these mechanobiological mechanisms may open up promising therapeutical options for the prevention and treatment of DDD.

## 5. Conclusions

The mechanobiology of the human intervertebral disc is a key element in degenerative disc disease. The technical advances that occurred in the last decade have allowed us to increase our understanding of this fascinating research topic, also enabling us to identify possible therapeutic targets in order to treat and to prevent DDD. Despite this, multiple pathways are involved in a complex and interplayed system, which is still partly unknown. Further research and technological innovation will further shed light on the interactions between the mechanics and biology of the human IVD.

## Figures and Tables

**Table 1:** Details of the included studies. ↑ Increase of expression, ↓ decrease of expression, ↑↑ high increase of expression, ↓↓ high decrease of expression, ⊗ inhibition.

Author	Study Aim	Samples	Age (Mean ± sd) Gender	Cell Type	Experimental Model	Molecular Pathway Involved	Mechanical Stimulus Applied	Observed Response		
Song et al. 2022 [32]	Analysis of MMP-12, CTGF and collagen I, III transcriptional regulation to assess the role of RhoA/MRTF-A signaling pathway in the development of IDD	NP tissues from lumbar spine of 25 human patients 15 patients with IVD 10 control patients with spinal cord injury	IVD patients: 55.5 ± 5.1 years -5 males -10 females Control group: 39.2 ± 7.6 years -4 males -6 females	NP	Human NP cells cultured on 2D flexible silicone Elastomer substrate and stretched on a Flexcell apparatus	RhoA/ROCK2– MRTF-A	CASES: 15% cyclic strain at a frequency of 6 cycles/min (cycle consist of 3 s of stretching alternating with 3 s of relaxation). Cyclic stretching was performed for 24 h. (Hyperphysiological mechanical loading) CONTROLS: NO mechanical strain	*Cyclic tensile strain (CTS) of NP cells* ↑ Nuclear translocation of MRTF-A ↑ RhoA, ROCK2, MRTF-A, SRF, MMP-12, and CTGF ↓ Aggrecan and collagen II		
								*CTS in NP cells + MRTF-A inhibitor (CCG-1423)* ⊗ RhoA signaling inhibited ⊗ MRTF-A transcription inhibited ⊗ Aggrecan and collagen II downregulation inhibited ↓ SRF, MMP-12, and CTGF		
Sun et al. 2020 [33]	To evaluate the role of NCs produced exosomes in the homeostasis of intervertebral disc and their link with mechanical load and disc angiogenesis	NP tissues from 20 human patients 10 IDD patients 10 AIS patients	IDD patients: 46.2 years (34–68) AIS patients: 19.3 years (16–24)	NC and NP cells	NC cell 2D culture samples subjected to controllable compressive stress at 0 MPa, 0.5 MPa, and 1.0 MPa for 24 and 48 h	miR-140-5p– Wnt/b-catenin axis	0 MPa, 0.5 MPa, and 1.0 MPa for 24 and 48 h	/		
	
Cambria et al. 2020 [34]	To investigate the role of TRPV4 in human AF cells stretch-induced inflammation	9 human IVD biopsies retrieved from patients undergoing spinal surgery for disc herniation or DDD	56.1 ± 20.5 years -4 males -5 females	AF	2D cultures in chambers mounted on commercial stretching bioreactor (STB-140-10, STREX)	TRPV4 ion channels	CASES: 20% cyclic sinusoidal uniaxial strain, frequency 1 Hz, cells were stretched for 1,2,4,8,12 and 24 h. (Hyperphysiological mechanical loading) CONTROLS: same condition without strain	*CTS in AF cells* ↑ IL-6, IL-8, COX2 and MMP-1. PGE2 release ↑ p38 and ERK phosphorylation (phosphorylated MAPKs) ↓ Aggrecan and collagen II Activation of p38		
								*CTS in AF cells + pharmacological inhibition of TRPV4* ⊗ p38 stretch-induced phosphorylation is inhibited		
								*CTS in AF cells + CRISPR-Cas9 Knock-Out of TRPV4* ⊗ IL-8, IL-6 and MMP-1 increase inhibited No changes in gene expression of COX2		
Franco-Obregon et al. 2018 [35]	To evaluate the effects of simulated microgravity and pharmacological inhibition of TRPC channel on human IVD cells	16 human IVD cells retrieved from surgical biopsies composed of both AF and NP 15 patients affected by DDD in the lumbar spine 1 patient affect by DDD in the cervical spine	51.5 ± 10.5 years -5 males -8 females -3 unknown	Both NP and AF	2D Culture chambers mounted on gimbal frames that simulated microgravity	TRPC6	Simulated microgravity (Hypophysiological mechanical loading)	↓ Cell proliferation ↑ Cell senescence and accumulation of cells in G2/M		
Hodson et al. 2018. [36]	To evaluate the role of mechanical and chemical IVD microenvironment on disc cells	NP cells from 3 human patients 2 human cadavers (IVD) 1 patient who underwent spinal surgery following trauma of the spine	Cadavers: 2 males aged 36 and 63 Spinal surgery patient: female aged 47	NP	Precultured NP cells embedded in agarose constructs and compressed using a Flexcell FX4000 Compression System (Flexcell International)	Catabolic and anabolic genes (multiple genes, mainly aggrecan and MMP-3)	CASES: 0.004 MPa, 1.0 Hz and 1 h of compression under pH conditions representative of nondegenerate (pH 7.1) and degenerate (pH 6.5) IVDs (Physiological mechanical loading) CONTROLS: uncompressed	*Non-degenerative IVD conditions–pH (7.1)* ↑ AGC, TIMP1, MMP-3, ADAMTS5 (increase in both anabolic and catabolic gene expression) AGC expression is modulated by integrin receptors, the presence of RGD peptides prevented the AGC expression increasing		
								*Degenerative IVD conditions–pH (6.5)* ↑ MMP-3 (loss of mechanoregulation of anabolic gene expression) AGC gene expression was not mechanoresponsive at pH 6.5. The increase in MMP-3 expression did not appear to be RGD-integrin dependent		
Likhitpanichkul et al. 2016 [37]	To assess the interaction between TNF-α and tensile strain levels on AF cells in vitro. To test the effectiveness of anti-TNF-α drug and anti-IL-6 drug on AF cells	AF cells from 5 human IVD derived from spinal surgeries and autopsies	50 ± 16 years	AF	AF cells 2D cultures in silicone-membrane chambers (STREX, B-Bridge, Cupertino, CA)	Pro-inflammatory cytokines (TNF-α, IL-1β, IL-8 and IL-6) and cytoskeletal changes (F-actin, vinculin, and α-tubulin)	CASES: TNF-α + physiological (5%) and pathological (15%) cyclic tensile uniaxial strain at 0.5 hz for 24 h CONTROLS: unstrained	↑ IL-1β and IL-8 at 15% compared to 5% strain. ↑ F-actin ↑ Cell roundedness ↓ Microtubule network (α-tubulin) Infliximab non- significantly decreased IL-1β and IL-8 levels compared to TNF-α stimulated AF cells undergoing 15% strain, and significantly decreased IL-1β and IL 8 levels compared to anti-IL-6 treatment		
Gilbert et al. 2013 [38]	To investigate the role of integrins in the mechanoresponse of human AF cells	6 human AF samples derived from surgical procedures for DDD and autopsies 3 classified as degenerative 3 classified as non-degenerative	Degenerative samples: 41 years (29–49) Non-degenerative samples: 47 years (37–57)	AF	2D cultures on Silicone membranes stretched by using the FX4000 Flexercell Tension System (Flexcell International, Hillsborough, NC, USA))	MMP-3, ADAMTS-4, aggrecan and collagen I	CASES: cyclic tensile strain with and without function blocking RGD –peptides with 10% strain, 1.0 Hz for 20 min (Physiological mechanical loading) CONTROLS: unstrained	RAD or RGD peptide treatment in unstrained cells had no effect on gene expression		
								*CTS in non-degenerative AF cells + RAD peptides* ↓ ADAMTS-4 ↑ FAK phosphorylation No effect on collagen I gene expression		*CTS in degenerative AF cells + RAD peptides* ↓ Collagen I No effect on ADAMTS-4 gene expression No effect on levels of FAK phosphorylation
								*CTS in non-degenerative AF cells + RGD peptides* ↑ ADAMTS-4 ⊗ CTS induced increase in FAK phosphorylation is inhibited No effect on collagen I gene expression		*CTS in degenerative AF cells + RGD peptides* ↓ Collagen I No effect on ADAMTS-4 gene expression No effect on levels of FAK phosphorylation
Gilbert et al. 2011 [39]	To investigate the role of IL-1 and IL-4 in mechanotransduction. of human AF cell To evaluate the role of proinflammatory cytokines in the different cellular responses of non-degenerative and degenerative AF cells.	6 human AF samples 3 classified as degenerative (2 from patients who underwent spinal surgeries for DDD and 1 from cadaver, respectively) 3 postmortem non-degenerative	Degenerative samples: 50 years (29–66) Non-degenerative samples: 47 years (37–57)	AF	Silicone membranes within the Bioflex culture plates using the FX4000 Flexercell Tension System (Flexcell International)	MMP-3, ADAMTS-4, aggrecan and collagen I	CASES: cyclic tensile strain with and without IL-1Ra or IL-4RAb with 10% strain, 1.0 Hz for 20 min (Physiological mechanical loading) CONTROLS: unstrained	*CTS in non-degenerative AF cells + IL-1Ra * ↑ ↑ MMP-3		*CTS in degenerative AF cells + IL-1Ra* ↓↓ Collagen I
								*CTS in non-degenerative AF cells + IL-4RAb* ↓ MMP-3		*CTS in degenerative AF cells + IL-4RAb* ↓↓ Collagen I
								*CTS in non-degenerative AF cells without IL-1Ra or IL-4Rab* ↓ ADAMTS-4 and MMP-3 No change in the relative gene expression of aggrecan or collagen I		*CTS in degenerative AF cells without IL-1Ra or IL-4RAb* ↓ Aggrecan and collagen I No change in the relative gene expression of MMP-3 or ADAMTS-4
Huang et al. 2011 [40]	To investigate the role of ADAMTSs and TIMP-3 in NP cells under mechanical stress.	30 human NP samples 15 patients with DDD 15 patients with idiopathic scoliosis (controls)	Patients with DDD: 30.2 years (27–28) Patients with idiopathic scoliosis: 18.7 years (16–24)	NP	NP tissue explants stimulated in BioPress compression culture plates using the Flexercell Compression System (FX-4000C; Flexcell International)	ADAMTSs, aggrecan and TIMP-3	CASES: Compressive load of 0.35–0.95 MPa at 1 Hz for 2 h twice a day for 7 days. 1 h prior to load, samples were treated with or without TIMP-3 (Physiological mechanical loading) CONTROLS: uncompressed	*Compressive loading without TIMP-3* ↑ ADAMTS-1, 4, and 5 ↓ Aggrecan		
								*Compressive loading + TIMP-3* ↑↑ Aggrecan		
Gilbert et al. 2010 [41]	To investigate the effect of CTS on AF cells derived from both degenerative and non-degenerative IVDs	6 human AF samples 2 surgical samples classified as degenerative 4 postmortem samples classified as non-degenerative	Degenerative: 2 patients aged 29 and 66 Non-degenerative: 49.3 years (37–57)	AF	2D cultures of AF cells on Silicone membranes stretched by the FX4000 Flexercell Tension System (Flexcell International)	MMP-3, ADAMTS-4, aggrecan and collagen I	CASES: application of 20% CTS for 20 min at a frequency of 1.0 Hz or 0.33 Hz (Physiological and less than physiological mechanical loading frequency respectively) CONTROLS: unstrained	*CTS at 1 Hz in non-degenerative AF cells* ↓ ADAMTS-4 and MMP-3		*CTS at 0.33 Hz in non-degenerative AF cells* ↓ Collagen I and II
								*CTS at 1 Hz in degenerative AF cells* ↓ Aggrecan and Collagen I		*CTS at 0.33 Hz in degenerative AF cells* ↓ Collagen I, II and aggrecan
Neidlinger-Wilke et al. 2009 [42]	To assess the effects of mechanical load on expression of pleiotropin (PTN) and aggrecan in IVD cells and to determine the factors influencing endothelial cells migration.	Human IVD cells isolated from discs of 11 patients 8 disc degenerations 3 underwent surgery for trauma 1 scoliosis	44.1 ± 18.3 years -6 males -5 females	Both NP and AF	Cells embedded in collagen gels exposed to strain (AF) or hydrostatic pressure (NP)	PTN, aggrecan	CASES: 4% CTS at 1 Hz for AF cells (*n* 6). Hydrostatic pressure (0.25 or 2.5 MPa; 0.1 Hz) for NP cells (*n* 11). (Physiological mechanical loading) CONTROLS: unstrained/unloaded	*CTS on AF cells for 1 h* ↑↑ PTN		*Hydrostatic pressure on NP cells for 1 h* ↑ PTN
								*CTS on AF cells for 24 h* ↑↑ Aggrecan No difference in PTN expression between loaded and unloaded cells		*Hydrostatic pressure on NP cells for 24 h* ↓ Aggrecan ↑ Endothelial cell migration/ adhesion No difference in PTN expression between loaded and unloaded cells
Le Maitre et al. 2009 [43]	To assess the role of integrin signaling in IVD cells exposed to mechanical stimulation.	24 human IVD samples 15 surgical samples classified as degenerative 9 postmortem samples classified as non-degenerative	Degenerative patients: 56.2 ± 15.8 years -10 males -4 females -1 unknown Non-degenerative patients: 43.8 ± 8.5 years -6 males	NP	NP cells seeded in alginate constructs loaded in BioFlex compression plates and stimulated with a Flexercell FX-4000 C compression loading system (Flexcell International)	Aggrecan	CASES: compression loading at 0.35–0.95 MPa at 1 Hz applied for 2 h. 1 h prior to mechanical loading, constructs were either untreated, treated with RGD integrins inhibitory peptide or with a control peptide. (Physiological mechanical loading) CONTROLS: uncompressed	*Compressive loading of degenerative and non-degenerative NP cells + control peptide* ↓ Aggrecan		*Compressive loading of non-degenerative NP cells + RGD-integrin ligand site inhibitor* ⊗ load-induced decrease in expression of aggrecan gene.
										*Compressive loading of degenerative NP cells + RGD-integrin ligand site inhibitor* ↓ Aggrecan
Le Maitre et al. 2008 [44]	To investigate the effect of physiological hydrostatic pressure on NP and AF cells.	Human IVD cells isolated from discs of 15 patients 7 surgical 8 postmortem	Surgical samples: 43.8 ± 15.6 -6 males -1 female Postmortem samples 74.8 ± 0.5 -8 males	NP	Alginate NP cell cultures in Sterile Whirl-Pak bags placed within a pressure vessel attached to a piston	Catabolic and anabolic genes (multiple genes, mainly aggrecan and MMP-3)	CASES: dynamic hydrostatic pressure at 0.8–1.7 MPa at 0,5 Hz for 2 h. (Physiological mechanical loading) CONTROLS: uncompressed	*HP on degenerative and non-degenerative AF cells* No effect on gene expression		
								*HP on non-degenerative NP cells* ↑↑ Aggrecan, c-fos ↑ Sox-9, collagen II No effect on MMP-3		*HP on degenerative NP cells* No effect on gene expression
Aladin et al. 2007 [45]	To compare the mechanical properties of human NP tissue in those that carry the Trp2 allele to those without, before any sign of disc degeneration.	12 non-degenerated IVD samples from patients who underwent scoliosis surgery (6 Trp2+ samples and 6 Trp2- samples). 6 degenerated IVD samples from patients who underwent anterior discectomy (Trp2-).	Non-degenerated samples (Trp2+): mean age 15.2 years Non degenerated samples (Trp2-): mean age 18.5 years Degenerated samples mean age 37.67 years	NP	Frozen tissue explants stimulated in a compression apparatus composed of non-porous chamber made of Teflon, placed coaxially to an upper rigid and porous platen having 50% porosity with an average pore size of 50 mm	COL9A2 Trp2 allele	NP samples were tested in confined compression. Swelling pressure and compressive moduli were measured and compared between groups (harboring or not the COL9 Trp2 allele)	/		
	
Neidlinger-Wilke et al. 2005 [46]	To evaluate the response of IVD cells, in AP and NF cells as well, to different mechanical stimuli in terms of mRNA expression of anabolic and catabolic matrix proteins	Human IVD cells isolated from discs of 18 patients surgically treated for DDD	41.1 ± 12.0 years -6 males -5 females	Both NP and AF	2D cultures in Silicone dishes (cyclic strain experiments) or into standard culture dishes (hydrostatic pressure experiments)	Catabolic and anabolic genes (multiple genes, mainly aggrecan and MMP-3)	STRAIN CASES: CTS of 1, 2, 4, and 8% at a frequency of 1 Hz for 24 h HYDROSTATIC PRESSURE CASES: 0.25 MPa at a frequency of 0.1 Hz for 30 min (Physiological mechanical loading) CONTROLS: unstrained and uncompressed	*HP on NP cells* ↑ Proliferation ↑ Aggrecan, collagen I ↓ MMP-2, MMP-3		*NP cells without HP or CTS* ↑ Proliferation
								*HP on AF cells* ↑ Collagen I ↓ MMP-3, aggrecan		
								*CTS on NP cells* ↑ Collagen I ↓ MMP-2, MMP-3		
								*CTS on AF cells* ↑ Aggrecan, collagen II ↓ MMP-3		

**Table 2:** Key findings of the included studies.

**Author**	**Key Findings**
Song et al. 2022 [32]	The RhoA/MRTF-A signaling pathway is activated by mechanical stress and promotes ECM degeneration in the NP. The RhoA/MRTF-A axis regulates MMP-12 and CTGF expression. NP degeneration caused by mechanical stress overload is mitigated by the RhoA/MRTF-A inhibitor CCG-1423, which has the potential therapeutic effect of NPCs’ functional recovery facilitation.
Sun et al. 2020 [33]	HUVECs’ migration is strongly inhibited by 0.5 Mpa/NC-exos, showing a dose-dependent effect. Thus, NC-exos induced by compressive load have the potential to inhibit endothelial cell angiogenesis. The anti-angiogenic role of 0.5 Mpa/NC-exos appears to be mediated by miRNAs transferred from NCs to endothelial cells via NC-exos. Among these miRNAs, the upregulation of miR-140-5p shows a significant inhibitive effect on angiogenesis in endothelial cells via the downregulation of Wnt11 expression and inhibition of b-catenin nuclear accumulation.
Cambria et al. 2020 [34]	A TRPV4 antagonist has shown the capacity to lessen the mechanoflammation caused by hyperphysiological CTS, thus revealing the novel mechanoinflammatory role of TRPV4 in human primary AF cells. TRPV4 mediates stretch-induced inflammation, possibly via the activation of the p38 MAPK pathway. TRPV4 KO via CRISPR-Cas9 prevents the stretch-induced upregulation of IL8 mRNA and tends to reduce the stretch-induced IL6 mRNA.
Franco-Obregon et al. 2018 [35]	IVD cells exposed to simulated microgravity or to TRPC channel inhibition showed reduced proliferation and increased senescence. The TRPC6 gene expression was reduced in cells subjected to simulated microgravity. Mechanotransduction, cell proliferation and senescence regulation in IVDs may be partially controlled by TRPC6 ion channels.
Hodson et al. 2018 [36]	NP cells cultured both at pH 7.1 and 6.5 showed similar gene expression patterns. NP cells’ response to compression shifted from matrix homeostasis at a pH of 7.1, representative of non-degenerated tissue, to matrix catabolism at a pH of 6.5, representative of degenerated tissue. Thus, a catabolic shift in human NP cell phenotype occurred under acidic pH conditions. The aberrant mechanotransduction induced by an acidic environment may have a potential role in the progression of IVD degeneration.
Likhitpanichkul et al. 2016 [37]	TNF-α-treated AF cells showed increased IL-1β and IL-8 production and cytoskeletal network modification with enhanced stress fiber formation from actin polymerization and microtubule network disruption; these cytoskeletal changes reflect increased mechanosensitivity. Pro-inflammatory cytokines produced by IVD cells may be a key factor in the inflamed environment associated with IVD degeneration, potentially altering physiological IVD mechanobiology.
Gilbert et al. 2013 [38]	RGD-recognizing integrins may not be involved in the altered mechanoresponse of AF cells derived from degenerate IVDs. The aberrant response in degenerate cells may be recognized by different mechanoreceptors via an alternative mechanotransduction pathway. The involvement of RGD integrins in mechanoresponses appeared to be lost with degeneration. The findings suggested FAK activation is caused by CTS via an RGD-integrin-dependent pathway. The ADAMTS-4 decreased gene expression seen in CTS-stimulated non-degenerated AF cells may occur through an integrin-, and potentially FAK-, dependent mechanism. Non-degenerated AF cells’ mechanoresponse to CTS appears to be mediated by RGD-recognizing integrins. The aforementioned cells showed the integrin-dependent phosphorylation of FAK in response to strain stimulation. RGD integrins and FAK may not be involved in the mechanoresponse of degenerated AF cells.
Gilbert et al. 2011 [39]	For the first time, immunopositivity for the IL-4r subunits IL-4Ra and IL2Rg and immunonegativity for the receptor subunit IL-13Ra1 was described in human IVD cells. Stimulated with CTS, AF cells derived from non-degenerative IVDs showed a shift to a less catabolic phenotype. The reduced catabolic response appeared to be IL-1- and IL-4-dependent. The increment in MMP-3 gene expression may be correlated with its decreased activity (negative feedback regulation). The inhibition of MMPs’ activity due to IL-1Ra treatment seems to have determined increased MMP gene expression in IVD cells, outlining the potential of targeting IL-1R in DDD treatment. The findings show the potential role of IL-1, not only in a catabolic shift typical of DDD, but also in transducing physiological mechanical stimuli, leading to tissue remodeling. Neither IL-1 nor IL-4 appeared to be necessary for the matrix remodeling mechanoresponse of AF cells derived from degenerative IVDs. AF cells derived from degenerative and non-degenerative tissue regulated matrix protein and matrix-degrading enzyme gene expression differently; this difference may be determined by altered cytokine-dependent transcription factor activation.
Huang et al. 2011 [40]	Compressive load, with the increase in ADAMTS-1, 4, 5 expression, appears to be a key factor in aggrecan depletion in human NP cells, which might have an etiologic implication in the development of IDD. The physiological expression of ADAMTS-1, 4, 9, 15 and TIMP-3 in non-degenerative discs indicates a possible role for these proteins in the normal turn-over of aggrecan and other matrix molecules in the healthy disc matrix. The imbalance between ADAMTSs and their inhibitor (TIMP-3) could play a role in the pathogenesis of IDD. TIMP-3 might be a potent therapeutic target preventing aggrecan loss during IDD.
Gilbert et al. 2010 [41]	The response of AF cells derived from both degenerative and non-degenerative IVDs to CTS appeared to be frequency-dependent. At 1 Hz of CTS (similar to normal physiologic motion), AF cells derived from non-degenerative tissue showed a shift to a less catabolic phenotype. The 0.33 Hz of CTS resulted in a catabolic response. The response of AF cells to CTS was both frequency-dependent and dependent on whether the cells are derived from degenerated or non -degenerated tissue. AF cells derived from degenerated tissue not only lost their ability to downregulate matrix enzyme gene expression in response to a 1.0 Hz frequency of CTS but also responded by reducing their matrix protein gene expression. Matrix homeostasis and matrix anabolism are promoted by physiological mechanical loads in healthy disc cells. Frequencies below physiological levels could lead to the degradation of the IVD matrix. On the contrary, physiologic mechanical loads could be detrimental to disc cell matrix homeostasis in degenerated IVDs, potentially leading to the progression of DDD.
Neidlinger-Wilke et al. 2009 [42]	Relative expression levels of PTN in human IVD cells are influenced by mechanical load. Increased PTN expression may have pro-angiogenic effects, which could be either enforced or impaired by the down- or upregulation of the antiangiogenic protein aggrecan. PTN expression showed high variability between different patients; this variation may be attributable to different degrees of disc degeneration. Mechanical load influences the presence of angiostatic and angiogenic factors within the IVD.
Le Maitre et al. 2009 [43]	In non-degenerated IVDs, RGD-binding integrins contribute to the mechanotransduction response. Mechanotransduction pathways appeared to be altered in NP cells derived from degenerative IVDs. Mechanosensing in NP cells from non-degenerative discs occurred via RGD integrins, possibly via the a5b1-integrin, while cells from degenerated discs might use a different receptor to sense and respond to mechanical signaling. Since the a5b1 integrin is physiologically expressed in degenerated IVDs, with no change in expression observed during disc degeneration, the cited mechanosensing could occur through a non-RGD integrin.
Le Maitre et al. 2008 [44]	IVD cells modify matrix homeostasis in response to physiological hydrostatic pressure. Cells obtained from healthy IVD tissues respond to dynamic HP by upregulating the gene expression of anabolic genes, indicative of healthy matrix homeostasis. Altered mechanotransduction pathways may be operational in degenerative IVD tissues.
Aladin et al. 2007 [45]	A non-degenerated (COL9 A2 Trp2+) control group appeared to have an increasing swelling stress pattern. Degenerated (COL9 A2Trp2-) groups exhibited decreasing swelling stress patterns. The non-degenerated (Trp2+) and degenerated (Trp2-) groups’ swelling pressure and compressive modulus were considerably lower than those of the non-degenerated (Trp2-) controls. A significant difference occurred in the swelling pressure (*p* = 0.006) and compressive modulus (*p* = 0.025) between the non-degenerated (Trp2-) controls and (Trp2+) cases. The swelling pressure difference between degenerated (Trp2+) controls and (Trp2-) cases was not significant (*p* = 0.34), suggesting that the Trp2 allele is related with a decrease in the swelling pressure to an extent approximately similar to but slightly better than that of the degenerated discs. The Trp2 allele seems to be linked to IVD mechanical properties and consequently to disc degeneration pathomechanism. Alterations in collagen IX result in cross-linking abnormalities between collagen IX and collagen II, thus reducing the ability of the collagen network to hold the ECM together, manifested as changes in the compression modulus and swelling pressure. The Trp2 allele is associated with deteriorating mechanical properties of the nucleus pulposus as early as adolescence, with no other signs of disc degeneration.
Neidlinger-Wilke et al. 2005 [46]	CTS appeared to have an anabolic effect both in AF and NP cells, apparently without differences among different strain magnitudes. The application of mechanical stimuli had no negative effect on IVD cells. Hydrostatic pressure also appeared to determine an anabolic effect in both AP and NP cells. Anulus cells were more responsive to cyclic strain, whereas nucleus cells were more responsive to hydrostatic pressure. Mechanically stimulated disc cells, either subjected to hydrostatic pressure or cyclic strain, tended to decrease the mRNA expression of MMP-2 and -3. These findings seem to suggest that moderate mechanical loading may prevent disc matrix degradation via the suppression of cellular production of MMPs. Cells obtained from different donor patients showed high inter-individual variations, which could be influenced by the degree of degeneration of the disc samples.

## Data Availability

Not applicable.
